# Peripheral blood metabolic composite score based on peripheral blood metabolism can be used as an assessment of recurrence after surgery in patients with locally advanced gastric cancer: a novel and promising index

**DOI:** 10.3389/fonc.2025.1536811

**Published:** 2025-04-17

**Authors:** Ning Meng, Zhiqiang Wang, Yaqi Peng, Xiaoyan Wang, Wenju Yue, Le Wang, Jingxia Lv, Wenqian Ma

**Affiliations:** ^1^ Department of General Surgery, Shijiazhuang People’s Hospital, Shijiazhuang, Hebei, China; ^2^ Basic College, Hebei Medical University, Shijiazhuang, Hebei, China; ^3^ The Third Department of Surgery, The Fourth Hospital of Hebei Medical University, Shijiazhuang, China; ^4^ Hebei Key Laboratory of Precision Diagnosis and Comprehensive Treatment of Gastric Cancer, The Fourth Hospital of Hebei Medical University, Shijiazhuang, Hebei, China; ^5^ Big Data Analysis and Mining Application for Precise Diagnosis and Treatment of Gastric Cancer, Hebei Provincial Engineering Research Center, Shijiazhuang, China; ^6^ Department of Endoscopy, The Fourth Hospital of Hebei Medical University, Shijiazhuang, China

**Keywords:** locally advanced gastric cancer, postoperative recurrence, metabolic markers, predictive model, nomogram

## Abstract

**Background:**

Postoperative recurrence remains a major challenge in patients with locally advanced gastric cancer (LAGC). Identifying reliable biomarkers for predicting recurrence can guide clinical decision-making and improve patient outcomes. This study aimed to investigate the association between four peripheral blood metabolic markers and postoperative recurrence in LAGC patients, and to develop a predictive model based on these markers.

**Methods:**

This retrospective cohort study analyzed data from 1,040 patients with LAGC who underwent radical surgical resection between January 2010 and December 2019. Peripheral blood metabolic indicators, including low-density lipoprotein/high-density lipoprotein (LHR), cholesterol/high-density lipoprotein (TCHR), triglycerides/high-density lipoprotein (TGHR), and triglycerides × fasting blood glucose (TyG), were used to assess metabolic status. Multivariable regression and survival analysis were performed to assess the prognostic value of these markers. A nomogram combining metabolic markers and clinical factors was developed and validated for predicting postoperative recurrence.

**Results:**

High levels of LHR, TCHR, TGHR, and TyG were significantly associated with increased risk of postoperative recurrence in LAGC patients (P < 0.001). Multivariable analysis identified TNM stage, pathological type, systemic immune inflammation index (SII), and metabolic score as independent predictors of recurrence. A predictive model incorporating these factors demonstrated superior performance compared to clinical features alone, with an area under the curve (AUC) of 0.867 (95% CI: 0.836-0.897) in the training set, 0.887 (95% CI: 0.844-0.929) in internal validation set, 0.859 (95% CI: 0.817-0.899) in the external validation set. Patients with high metabolic scores had significantly worse overall survival (OS) and disease-free survival (DFS), further supporting the model’s prognostic value.

**Conclusions:**

Peripheral blood metabolic markers, particularly LHR, TCHR, TGHR, and TyG, are valuable predictors of postoperative recurrence in LAGC patients. The combined predictive model, integrating metabolic markers and clinical features, provides an effective tool for personalized risk stratification and may assist in optimizing postoperative management in LAGC.

## Introduction

Locally advanced gastric cancer (LAGC) is associated with a high risk of postoperative recurrence, which significantly impacts patient survival (1, 2). Despite advancements in surgical techniques and adjuvant therapies, predicting recurrence in LAGC patients remains a significant challenge (3). Current methods of recurrence prediction mainly rely on clinical staging and conventional biomarkers, such as tumor size, lymph node involvement, and serum markers (e.g., carcinoembryonic antigen, CEA) (4, 5). While these tools provide valuable information, they often lack sufficient sensitivity and specificity to accurately identify high-risk patients, particularly in the early postoperative period when timely intervention can have the most significant impact on outcomes.

Recent research has increasingly focused on the role of metabolic dysregulation in cancer progression (6–8). Several studies have shown that alterations in metabolic pathways, such as lipid metabolism, glucose homeostasis, and insulin resistance, are associated with tumor progression and recurrence in various cancers, including gastric cancer (9–11). These metabolic changes reflect the underlying pathophysiological processes that support tumor growth and metastasis. As a result, metabolic biomarkers have gained attention as potential prognostic indicators for cancer recurrence. However, most studies have investigated individual metabolic markers, such as the lipid profile or the triglyceride-glucose index (TyG) in isolation (12–14). Previous studies have shown that LHR is associated with poor prognosis in colorectal cancer and gastric cancer (15, 16). While these markers have demonstrated some predictive value, they may not provide a complete picture of a patient’s metabolic status and its relationship with tumor behavior.

To address this gap, recent approaches have explored the integration of multiple metabolic indicators into a composite score, aiming to enhance the accuracy and reliability of recurrence prediction. By combining several metabolic markers, a composite score can more comprehensively reflect the complex metabolic changes that occur in cancer patients (17–20). This approach not only strengthens the predictive value but also offers the advantage of being based on widely accessible and routinely measured biomarkers, making it a practical tool for clinical use. Previous studies have highlighted the potential of combining lipid ratios with glucose-related indices to improve risk stratification in cancer patients.

This study aims to address the gap in current recurrence prediction methods by exploring the potential of a peripheral blood metabolic composite score to predict postoperative recurrence in LAGC patients. By combining several established metabolic markers, such as the Low-Density Lipoprotein/High-Density Lipoprotein Ratio (LHR), Total Cholesterol/High-Density Lipoprotein Ratio (TCHR), Triglyceride/High-Density Lipoprotein Ratio (TGHR), and the Triglyceride-Glucose Index (TyG) into a single score, we seek to develop a more robust and reliable tool for assessing recurrence risk. This approach offers the potential for more accurate risk stratification, allowing clinicians to identify high-risk patients and tailor postoperative surveillance and adjuvant treatment accordingly.

The novelty and significance of this study lie in its focus on integrating multiple metabolic markers into a single, composite score, which could offer a more accessible, cost-effective, and clinically applicable method for recurrence prediction. By leveraging commonly measured peripheral blood markers, our study aims to provide a practical solution for improving personalized care and outcomes in LAGC patients. Ultimately, we hope that this approach will lead to earlier interventions, better treatment planning, and improved survival rates in this challenging patient population.

## Methods

### Study population

This retrospective cohort study used data from the Hebei Gastric Cancer Collaborative Network database (http://hbss.suvalue.com/), which prospectively collects data on the diagnosis and treatment of gastric cancer (21, 22). The study included patients with LAGC who underwent radical surgical resection between January 2010 and December 2019. All patients were aged ≥18 years, had a diagnosis of adenocarcinoma confirmed by gastric biopsy, and had not received preoperative chemotherapy, radiotherapy, or other anticancer treatments. Inclusion was limited to patients with a hospital stay >48 hours. Exclusion criteria included patients with active infections or autoimmune diseases, as well as those with missing data (e.g., age, height, TNM staging, fasting blood glucose, LDL cholesterol, HDL cholesterol, total cholesterol, triglycerides [TG], neutrophil count, or lymphocyte count). The study adhered to the principles of the Declaration of Helsinki and was approved by the local ethics committee. Informed consent was obtained from all patients, either in writing or orally, with the understanding that their clinical data would be used without disclosing personal information.

A total of 1040 LAGC patients were classified into training and validation cohorts based on the medical center where they were treated. Patients were from the Fourth Hospital of Hebei Medical University(FHHM), and this group was randomly divided into a training cohort (n=515) and an internal validation (n=221) cohort in a 7:3 ratio via the “caret” package in R software. Patients from Shijiazhuang People’s Hospital(SJZPH) were assigned to the external validation cohort (n=304).

### Data collection

Patient data, including age, gender, tumor type, stage, and smoking and alcohol history, CEA, CA19-9 were obtained from the electronic medical records. Clinical staging was performed according to the AJCC TNM staging system (8th edition). After overnight fasting, serum biomarkers, such as albumin, total cholesterol, TG, low-density lipoprotein (LDL), high-density lipoprotein (HDL), neutrophil count, lymphocyte count, and blood glucose levels were collected within 24 hours of admission. All measurements were standardized to minimize variations due to laboratory equipment.

### Definition of peripheral blood metabolic indicators

Four peripheral blood metabolic indicators were used to assess metabolic status: LHR (low-density lipoprotein cholesterol/high-density lipoprotein cholesterol), TCHR (cholesterol/high-density lipoprotein cholesterol), TGHR (triglycerides/high-density lipoprotein cholesterol), and TyG (Ln [TG (mg/dL) ×mg/(mg/dL)]/2) (23, 24). The optimal cutoff values for each indicator were determined using the maximum rank statistics method.

### Study observation endpoint

The primary endpoint of this study was postoperative recurrence. Overall survival (OS) was measured in months, defined as the time from admission to death or the last follow-up. Disease-free survival (DFS) was defined as the time from randomization to disease recurrence or death due to disease progression. Clinical outcome data were collected through regular follow-ups or via telephone.

### Statistical analysis

Continuous variables are presented as means eantandard deviation or medians with interquartile range (IQR), while categorical variables are expressed as counts and percentages (n, %). The comparison of continuous variables was performed using independent t-tests or non-parametric tests, and categorical variables were compared using chi-square tests or Fisher’s exact test. Covariates and potential confounders were selected based on prior studies. Univariate and multivariable Cox regression analyses were used to assess hazard ratios (HRs) and 95% confidence intervals (CIs) for key prognostic factors affecting overall survival. Subgroup and sensitivity analyses were also conducted. Kaplan-Meier curves and log-rank tests were employed to depict survival trends and compare survival rates between groups. The predictive value of different models for recurrence in LAGC patients was assessed using receiver operating characteristic (ROC) curves. A two-sided p-value < 0.05 was considered statistically significant. All statistical analyses were performed using R software version 4.1.1 (https://www.r-project.org/).

## Result

### Association between four peripheral blood metabolic markers and clinical-pathological features in patients with LAGC

**Figure 1:**
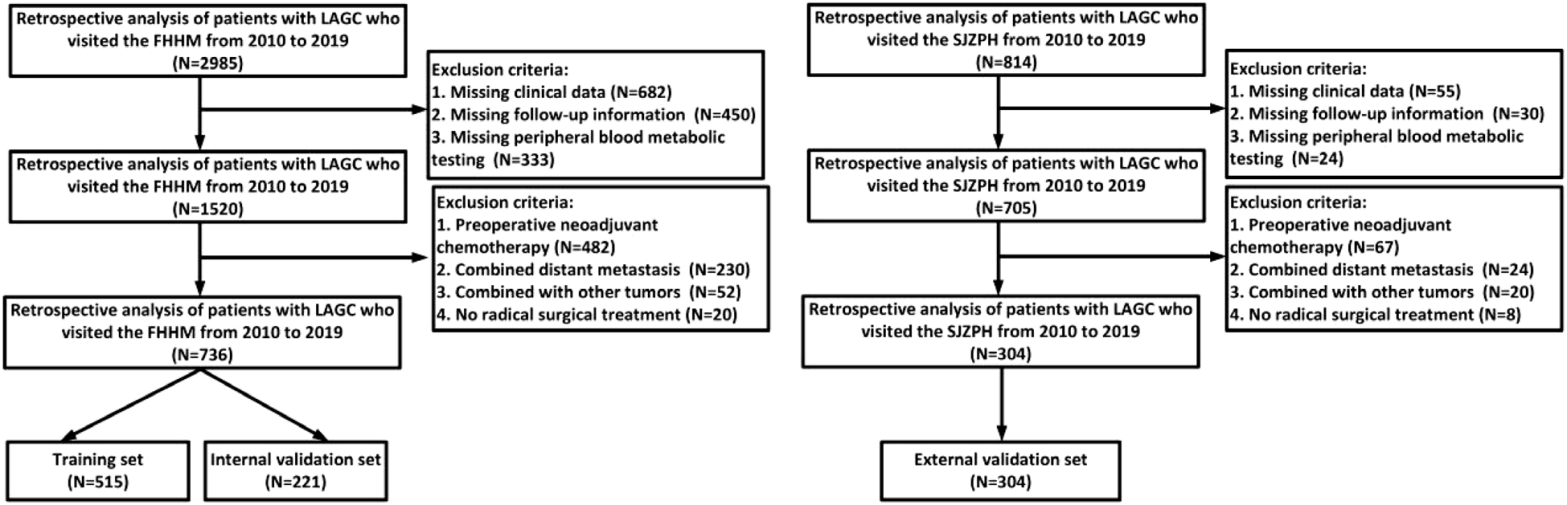
Flowchart of inclusion and exclusion of LAGC patients in this study.

This retrospective study analyzed data from 3799 patients diagnosed with LAGC at two medical centers between 2010 and 2019. After applying inclusion and exclusion criteria, 1040 patients were included in the final analysis (detailed screening process shown in **Figure 1**). **Table 1** illustrates the relationships between clinical characteristics and different metabolic marker groups (LHR, P, TCHR, and TyG). Regarding gender, 66.6% of males were in the LHR high group, and 66.7% of females were in the TyG low group. The proportion of females was higher in both the LHR low and TyG high groups (43.2% and 42.7%, respectively). Age distribution was similar between patients ≤65 years and >65 years, with no significant differences across groups (P > 0.05). In the TNM staging, most patients were classified as stage III (61.0%), with a higher concentration of stage III patients in the LHR high (51.9%) and TyG low (78.6%) groups.

**Table 1:** Relationship between peripheral blood metabolic indexes and clinicopathological characteristics of patients with gastric cancer.

Variable	Total (N=1040)	LHR		P	TCHR		P	TGHR		P	TyG		P
		High (N=464)	Low (N=576)		High (N=510)	Low (N=530)		High (N=474)	Low (N=566)		High (N=429)	Low (N=611)	
Gender				0.001			0.032			0.243			0.002
Male		309(66.6)	327(56.7)		295(57.8)	341(64.3)		299(63.1)	337(59.5)		286(66.7)	350(57.3)	
Female		155(33.4)	249(43.2)		215(42.1)	189(35.7)		175(36.9)	229(40.5)		143(33.3)	261(42.7)	
Age(years)				0.507			0.048			0.087			0.165
≤65		240(51.7)	286(49.7)		242(47.5)	284(53.6)		226(47.7)	300(53.0)		228(53.1)	298(48.8)	
>65		224(48.3)	290(50.3)		268(52.5)	246(46.4)		248(52.3)	266(47.0)		201(46.9)	313(51.2)	
TNM stage				0.000			0.000			0.000			0.000
I		46(9.9)	132(22.9)		67(13.1)	111(20.9)		49(10.3)	129(22.8)		21(4.9)	157(25.7)	
II		135(29.1)	145(25.2)		151(29.6)	129(24.3)		145(30.6)	135(23.9)		71(16.6)	209(34.2)	
III		283(61.0)	299(51.9)		292(57.3)	290(54.7)		280(59.1)	302(53.4)		337(78.6)	245(40.1)	
Primary site				0.002			0.485			0.000			0.476
Up 1/3		137(29.5)	207(35.9)		169(33.1)	175(33.0)		180(38.0)	164(29.0)		148(34.5)	196(32.1)	
Middle 1/3		128(27.6)	182(31.6)		144(28.2)	166(31.3)		101(21.3)	209(36.9)		131(30.5)	179(29.3)	
Lower 1/3		199(42.9)	187(32.5)		197(38.6)	189(35.7)		193(40.7)	193(34.1)		150(35.0)	236(38.6)	
Tumor size(cm)				0.038			0.007			0.493			0.866
≤5		191(41.2)	201(34.9)		171(33.5)	221(41.7)		184(38.8)	208(36.7)		163(38.0)	229(37.5)	
>5		273(58.8)	375(65.1)		339(66.5)	309(58.3)		290(61.2)	358(63.3)		266(62.0)	382(62.5)	
Histology				0.269			0.606			0.002			0.872
None/Low		409(88.1)	520(90.3)		453(88.8)	476(89.9)		439(92.6)	490(86.6)		384(89.5)	545(89.2)	
High		55(11.9)	56(9.7)		57(11.2)	54(10.2)		35(7.4)	76(13.4)		45(10.5)	66(10.8)	
Nerve invasion				0.000			0.000			0.906			0.000
Yes		335(72.2)	334(58.0)		366(71.8)	303(57.2)		304(64.1)	365(64.5)		341(79.5)	328(53.7)	
No		129(27.8)	242(42.0)		144(28.2)	227(42.8)		170(35.9)	201(35.5)		88(20.5)	283(46.3)	
Vascular invasion				0.982			0.308			0.706			0.003
Yes		242(52.2)	300(52.1)		274(53.7)	268(50.6)		244(51.5)	298(52.7)		292(68.1)	361(59.1)	
No		222(47.8)	276(47.9)		236(46.3)	262(49.4)		230(48.5)	268(47.3)		137(31.9)	250(40.9)	
Lauren				0.731			0.412			0.000			0.153
Intestinal		166(35.8)	212(36.8)		179(35.1)	199(37.5)		143(30.2)	235(41.5)		145(33.8)	233(38.1)	
Mixed		298(64.2)	364(63.2)		331(64.9)	331(62.5)		331(69.8)	331(58.5)		284(66.2)	378(61.9)	
CEA				0.039			0.357			0.320			0.827
<5		219(47.2)	235(40.8)		230(45.1)	224(42.3)		199(41.9)	255(45.1)		189(44.1)	265(43.4)	
≥6		245(52.8)	341(59.2)		280(54.9)	306(57.7)		275(58.1)	311(54.9)		240(55.9)	346(56.6)	
CA19-9				0.555			0.681			0.001			0.241
<30		251(54.1)	301(52.3)		274(53.7)	278(52.5)		279(58.9)	273(48.2)		237(55.2)	315(51.6)	
≥15		213(45.9)	275(47.7)		236(46.3)	252(47.5)		195(41.1)	293(51.8)		192(44.8)	296(48.4)	

For tumor location, the proportion of tumors located in the lower third was higher in the LHR low (38.6%) and TCHR high (38.6%) groups. Regarding tumor size, 58.8% of patients had tumors larger than 5 cm, with 65.1% of patients in the LHR high group having tumors larger than 5 cm. The incidence of nerve infiltration was higher overall (72.2%), with higher rates in the LHR high (58.0%) and TyG low (79.5%) groups. While no significant difference was found for vascular infiltration (P > 0.05), the proportion of vascular infiltration was higher in the TyG high group (68.1%). Regarding Lauren classification, 64.2% of patients had the mixed type, with similar proportions observed across the LHR and TyG groups. Overall, the metabolic markers LHR, P, TCHR, and TyG were associated with clinical features such as gender, TNM stage, tumor size, and infiltration, which may influence the clinical presentation and prognosis of patients.

### Comparison of clinical features across different datasets

There were no significant differences in clinical characteristics, including gender, age, TNM stage, tumor location, tumor size, histological type, and the presence of nerve or vascular invasionicalic the three datasets (training, internal validation, and external validation) (P > 0.05). Gender distribution was relatively balanced across the groups, with males comprising approximately 60% and females 40%. The proportion of patients aged 65 years or younger was similar across all groups. Most patients were classified as stage III in TNM staging, with similar proportions across the groups. Additionally, there were no significant differences in tumor location, size, histological type, or Lauren classification. The incidence of nerve and vascular invasion showed minimal variation between the datasets. Overall, the clinical features across the different datasets were consistent, with no significant differences observed. **Table 2** provides a detailed comparison of clinical and pathological features across the different datasets.

**Table 2:** Comparison of clinical characteristics of patients in the training set and validation set.

Variable	Training (N=515)	Internal validation (N=221)	External validation (N=304)	P
Gender				0.889
Male	317(61.6)	136(61.5)	181(59.5)	
Female	198(38.4)	85(38.5)	121(40.5)	
Age(years)				0.901
≤65	261(50.7)	109(49.3)	156(51.3)	
>65	254(49.3)	112(50.7)	148(48.7)	
TNM stage				0.892
I	81(15.7)	37(16.7)	60(19.7)	
II	150(29.1)	56(25.4)	74(24.4)	
III	284(55.2)	128(57.9)	170(55.9)	
Primary site				0.763
Up 1/3	168(32.6)	74(33.5)	102(33.6)	
Middle 1/3	151(29.3)	68(30.8)	91(29.9)	
Lower 1/3	196(38.1)	79(35.7)	111(36.5)	
Tumor size(cm)				0.440
≤5	204(39.6)	78(35.3)	110(36.2)	
>5	311(60.4)	143(64.7)	194(63.8)	
Histology				0.852
None/Low	462(89.7)	198(89.6)	269(88.5)	
High	53(10.3)	23(10.4)	35(11.5)	
Nerve invasion				0.569
Yes	329(63.9)	137(62.0)	183(60.2)	
No	186(36.1)	84(38.0)	121(39.8)	
Vascular invasion				0.415
Yes	273(53.0)	120(54.3)	149(49.0)	
No	242(47.0)	101(45.7)	155(51.0)	
Lauren				0.484
Intestinal	193(37.5)	83(37.6)	102(33.6)	
Mixed	322(62.5)	138(62.4)	202(66.4)	
Recurrence				0.528
Yes	218(42.3)	85(38.4)	131(43.1)	
No	297(57.6)	136(61.5)	173(56.9)	
CEA				0.262
<5	214(41.6)	99(44.8)	141(46.4)	
≥4	301(58.4)	122(55.2)	163(53.6)	
CA19-9				0.572
<30	267(51.8)	116(52.5)	169(55.6)	
≥69	248(48.2)	105(47.5)	135(44.4)	

### Development of a predictive model for postoperative recurrence in LAGC patients based on four peripheral blood metabolic markers

We first analyzed the impact of four metabolic markers on the postoperative recurrence of LAGC patients following radical surgery. Multivariable logistic regression analysis revealed that high levels of LHR, TCHR, TGHR, and TyG were significantly associated with recurrence. As shown in **Table 3**, in the training set, the OR for LHR, TCHR, TGHR, and TyG were 4.352 (95% CI: 2.847-6.650), 3.342 (95% CI: 2.165-5.160), 2.839 (95% CI: 1.853-4.350), and 4.245 (95% CI: 2.751-6.548), respectively, all with P-values < 0.001. In the internal validation set, LHR (OR = 3.616, 95% CI: 1.866-7.004), TCHR (OR = 3.568, 95% CI: 1.768-7.201), TGHR (OR = 3.605, 95% CI: 1.863-6.977), and TyG (OR = 4.273, 95% CI: 2.124-8.597) also showed significant associations (P < 0.001). In the external validation set, LHR (OR = 4.210, 95% CI: 2.424-7.313), TCHR (OR = 3.824, 95% CI: 2.188-6.683), TGHR (OR = 3.009, 95% CI: 1.731-5.230), and TyG (OR = 3.767, 95% CI: 2.143-6.620) similarly demonstrated strong predictive capabilities (P < 0.001). These findings suggest that these metabolic markers are independent predictors of gastric cancer recurrence, and based on this, we developed a comprehensive metabolic score using these four markers.

**Table 3:** Multivariable logistic regression analysis of peripheral blood metabolic markers and recurrence of locally advanced gastric cancer.

Training cohort			
Variables	OR	95%CI	P value
LHR (High vs. Low)	4.352	2.847-6.650	<0.001
TCHR (High vs. Low)	3.342	2.165-5.160	<0.001
TGHR (High vs. Low)	2.839	1.853-4.350	<0.001
TyG (High vs. Low)	4.245	2.751-6.548	<0.001

Internal validation set			
Variables	OR	95%CI	P value
LHR (High vs. Low)	3.616	1.866-7.004	<0.001
TCHR (High vs. Low)	3.568	1.768-7.201	<0.001
TGHR (High vs. Low)	3.605	1.863-6.977	<0.001
TyG (High vs. Low)	4.273	2.124-8.597	<0.001

External validation set			
Variables	OR	95%CI	P value
LHR (High vs. Low)	4.210	2.424-7.313	<0.001
TCHR (High vs. Low)	3.824	2.188-6.683	<0.001
TGHR (High vs. Low)	3.009	1.731-5.230	<0.001
TyG (High vs. Low)	3.767	2.143-6.620	<0.001

**Figure 2:**
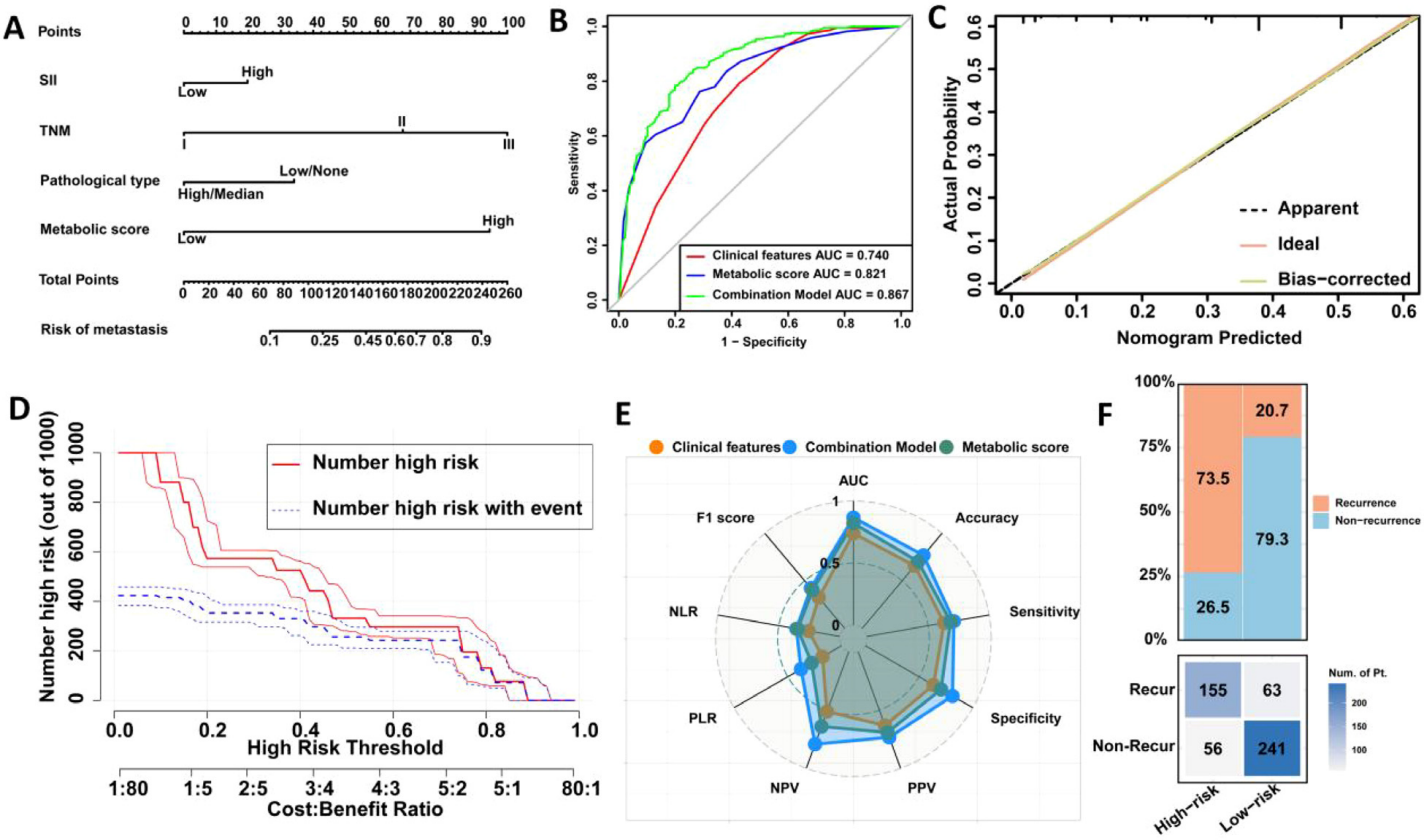
Construction and validation of a prediction model for postoperative recurrence in LAGC patients based on peripheral blood metabolic indicators. **(A)** Visual nomogram based on training set; **(B)** Comparison of ROC curve areas of different prediction models; **(C)** Calibration curve; **(D)** Clinical impact curve; **(E)** Radar chart; **(F)** Confusion matrix. AUC, Area under the curve; NLR, Negative Likelihood Ratio; PLR, Positive Likelihood Ratio; NPV, Negative Predictive Value; PPV, Positive Predictive Value; Recur, recurrence; Non-Recur, Non-recurrence.

Further multivariable analysis identified TNM stage, pathological type, SII, and metabolic score as significant predictors of postoperative recurrence in LAGC. In the training cohort, the risk of recurrence in stage III patients was 7.31 times higher than in stage I/II patients (OR = 7.312, 95% CI: 3.345-10.563, P < 0.001), while patients with high pathological types had a 2.24-fold increased risk compared to those with low or no pathological types (OR = 2.242, 95% CI: 1.323-4.672, P = 0.005). The recurrence risk for patients in the high SII group was 1.62 times higher than in the low SII group (OR = 1.623, 95% CI: 1.242-3.679, P = 0.010), and the risk for those in the high metabolic score group was 5.21 times higher than in the low score group (OR = 5.206, 95% CI: 3.633-9.220, P < 0.001) (**Table 4**). Based on these findings, we constructed a nomogram for predicting postoperative recurrence in LAGC patients (**Figure 2A**).

**Table 4:** Multivariable analysis of factors affecting recurrence in patients with locally advanced gastric cancer.

Training cohort			
Variables	OR	95%CI	P value
TNM stage (III vs. I/II)	7.312	3.345-10.563	<0.001
Pathological type (Low/None vs. High/Median)	2.242	1.323-4.672	0.005
SII (High vs. Low)	1.623	1.242-3.679	0.010
Metabolic score (High vs. Low)	5.206	3.633-9.220	<0.001

Internal validation set			
Variables	OR	95%CI	P value
TNM stage (III vs. I/II)	6.644	3.063-9.565	<0.001
Pathological type (Low/None vs. High/Median)	2.898	1.233-5.663	0.003
SII (High vs. Low)	1.677	1.234-3082	0.016
Metabolic score (High vs. Low)	4.784	2.231-6.765	<0.001

External validation set			
Variables	OR	95%CI	P value
TNM stage (III vs. I/II)	7.123	2.672-10.373	<0.001
Pathological type (Low/None vs. High/Median)	3.668	1.211-6.328	0.012
SII (High vs. Low)	1.855	1.349-4.789	0.022
Metabolic score (High vs. Low)	5.345	2.198-8.982	<0.001

ROC curve analysis indicated that the metabolic score model outperformed clinical features alone, with an AUC of 0.821 versus 0.740 (Delong test, p = <0.001) (**Figure 2B**). When combining clinical features with the metabolic score, the predictive AUC increased to 0.867 (95% CI: 0.836-0.897), significantly surpassing the performance of clinical features alone (0.867 vs. 0.740, Delong test, p = 0.001) or metabolic score alone (0.867 vs. 0.821, Delong test, p <0.001). Calibration curves further confirmed the strong predictive performance of the nomogram (**Figure 2C**). Clinical impact curve analysis demonstrated that the nomogram provided superior net benefits across a wide range of threshold probabilities, supporting the clinical value of the combined model (**Figure 2D**). Additionally, radar chart and confusion matrix analyses revealed that the combined model performed better than using clinical features or metabolic scores alone in the training set. The combined model in the training set achieved an AUC of 0.867, with an accuracy of 76.8%, sensitivity of 71.1%, and specificity of 81.1% (**Figures 2E, F**, **Table 5**).

**Table 5:** Comparison of performance indicators of different models for predicting recurrence of locally advanced gastric cancer.

Variable	AUC	DeLong test	Accuracy	Sensitivity	Specificity	PPV	NPV	PLR	NLR	F1 score
Training set										
Clinical features	0.740	<0.001	0.658	0.631	0.634	0.633	0.513	3.771	0.256	0.323
Metabolic score	0.821	<0.001	0.702	0.684	0.704	0.692	0.641	2.782	0.345	0.402
Combination Model	0.867	Reference	0.768	0.711	0.811	0.734	0.793	3.771	0.356	0.723
Internal validation set										
Clinical features	0.758	<0.001	0.642	0.677	0.567	0.301	0.9213	2.776	0.523	0.429
Metabolic score	0.814	<0.001	0.709	0.711	0.679	0.428	0.894	2.633	0.432	0.556
Combination Model	0.887	Reference	0.760	0.764	0.757	0.663	0.837	3.152	0.311	0.710
External validation set										
Clinical features	0.727	<0.001	0.650	0.686	0.673	0.485	0.768	1.434	0.549	0.466
Metabolic score	0.824	0.013	0.721	0.743	0.721	0.503	0.802	2.844	0.335	0.588
Combination Model	0.859	Reference	0.786	0.817	0.763	0.723	0.846	3.446	0.240	0.767
Training set										
Up 1/3										
Combination Model	0.868	/	0.774	0.803	0.753	0.704	0.839	3.24	0.262	0.750
Middle 1/3 and Lower 1/3										
Combination Model	0.866	/	0.804	0.776	0.825	0.765	0.833	4.43	0.272	0.770

### Validation of a predictive model for postoperative recurrence in LAGC patients based on four peripheral blood metabolic markers

We first analyzed the predictive performance of the model in the internal validation set. The OR for various factors were as follows: TNM stage (OR = 6.64, 95% CI: 3.063-9.565, P < 0.001), pathological type (OR = 2.90, 95% CI: 1.233-5.663, P = 0.003), SII (OR = 1.68, 95% CI: 1.234-3.082, P = 0.016), and metabolic score (OR = 4.78, 95% CI: 2.231-6.765, P < 0.001). In the external validation set, the OR values were: TNM stage (OR = 7.12, 95% CI: 2.672-10.373, P < 0.001), pathological type (OR = 3.67, 95% CI: 1.211-6.328, P = 0.012), SII (OR = 1.86, 95% CI: 1.349-4.789, P = 0.022), and metabolic score (OR = 5.35, 95% CI: 2.198-8.982, P < 0.001). These results demonstrate that TNM stage, pathological type, SII, and metabolic score are significant predictors of recurrence in locally advanced gastric cancer across both validation sets (**Tables 3**, **4**).

**Figure 3:**
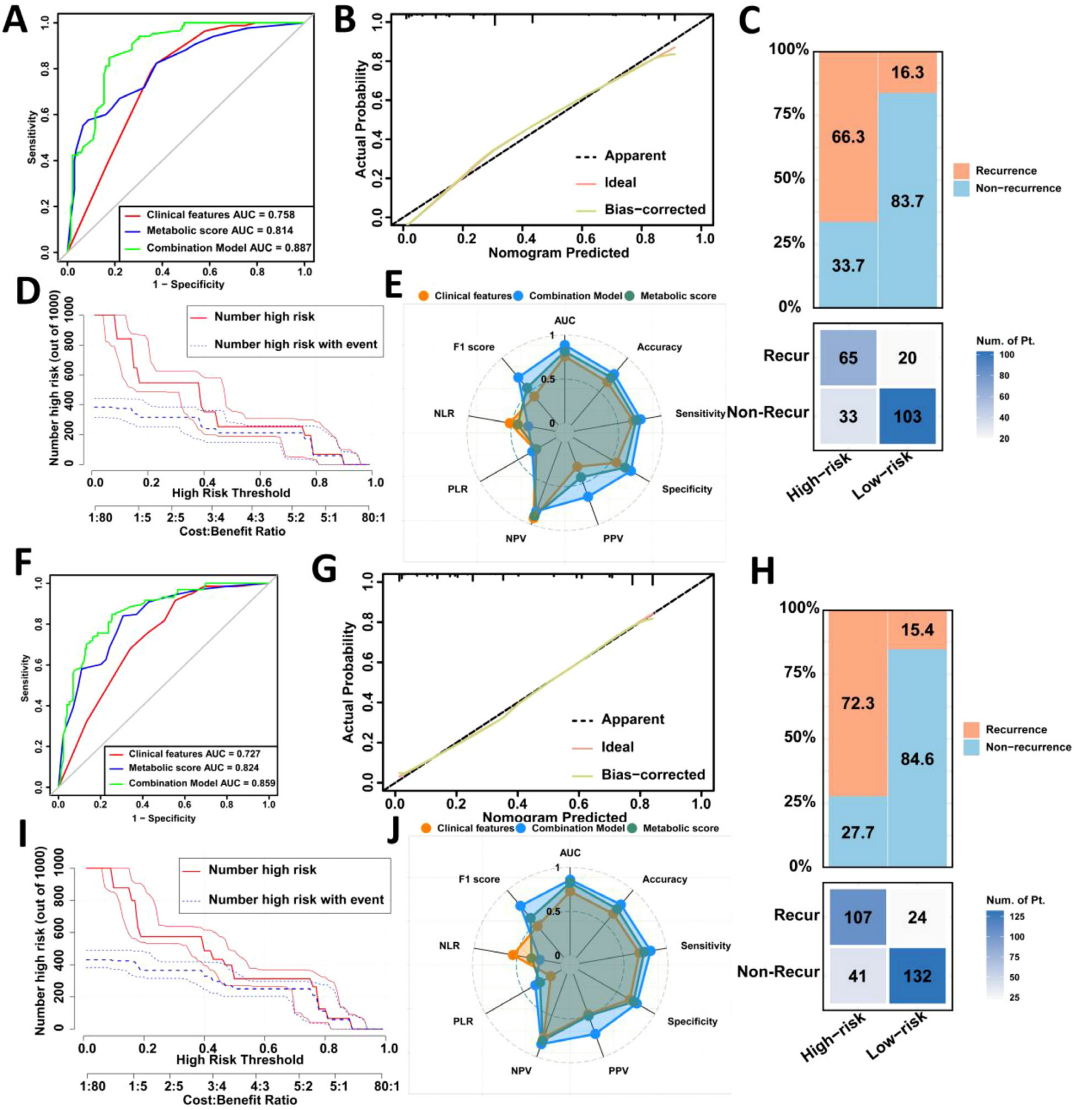
Validation of the prediction model for postoperative recurrence in LAGC patients in the internal validation set and the external validation set. **(A)** Comparison of ROC curve areas of different prediction models in the internal validation set; **(B)** Calibration curve in the internal validation set; **(C)** Confusion matrix in the internal validation set; **(D)** Clinical impact curve in the internal validation set; **(E)** Radar chart in the internal validation set; **(F)** Comparison of ROC curve areas of different prediction models in the external validation set; **(G)** Calibration curve in the external validation set; **(H)** Confusion matrix in the external validation set; **(I)** Clinical impact curve in the external validation set; **(J)** Radar chart in the external validation set. AUC, Area under the curve; NLR, Negative Likelihood Ratio; PLR, Positive Likelihood Ratio; NPV, Negative Predictive Value; PPV, Positive Predictive Value; Recur, recurrence; Non-Recur, Non-recurrence.

Next, we plotted ROC curves in the internal validation set, which revealed that the combined model outperformed both clinical features alone (AUC = 0.887 vs. 0.758, Delong test, p < 0.001) and metabolic score alone (AUC = 0.887 vs. 0.814, Delong test, p < 0.001) (**Figure 3A**). Similar results were observed in the external validation set (**Figure 3F**). Calibration curve analysis further confirmed the robust predictive performance of the combined model in both internal and external validation sets (**Figures 3B, G**). Clinical impact curve analysis showed that the nomogram provided superior net benefit over a wide range of threshold probabilities, indicating the significant predictive value of the combined model (**Figures 3D, I**).

Confusion matrix and radar chart analyses revealed that the combined model in the internal validation set achieved excellent results, with an AUC of 0.887, accuracy of 76.0%, sensitivity of 76.4%, and specificity of 75.7% (**Figures 3C, E**). In the external validation set, the combined model had an AUC of 0.859, accuracy of 78.6%, sensitivity of 81.7%, and specificity of 76.3%, outperforming single-factor models (**Figures 3H, J**).

Overall, the combined model demonstrated strong predictive capability across multiple performance metrics, particularly in terms of accuracy, sensitivity, and specificity, confirming the effectiveness of integrating clinical features and metabolic scores in predicting gastric cancer recurrence (**Table 5**).

### Comparison of predictive model for postoperative recurrence in LAGC patients based on before and after radical operation four peripheral blood metabolic markers

We collected serum biomarkers for patients within 7 days after surgery compared them with those before surgery, and the results showed no significant differences (**Supplementary Table 1**). Then we constructed a metabolic score model based on the four serum biomarkers within 7 days after surgery, and ROC curve analysis indicated no significant differences compared to the model using preoperative markers. Subsequently, we integrated clinical features with the metabolic score, with the ROC of 0.858 (0.826-0.889) in the training set, 0.874 (0.828-0.918) in internal validation set, 0.847 (0.804-0.891) in the external validation set. The Delong test results showed that in the train and external validation set, the model based on the preoperative serum biomarkers demonstrated better performance (**Supplementary Table 2**).

### Association between metabolic score based on four peripheral blood metabolic markers and prognosis in LAGC patients

**Figure 4:**
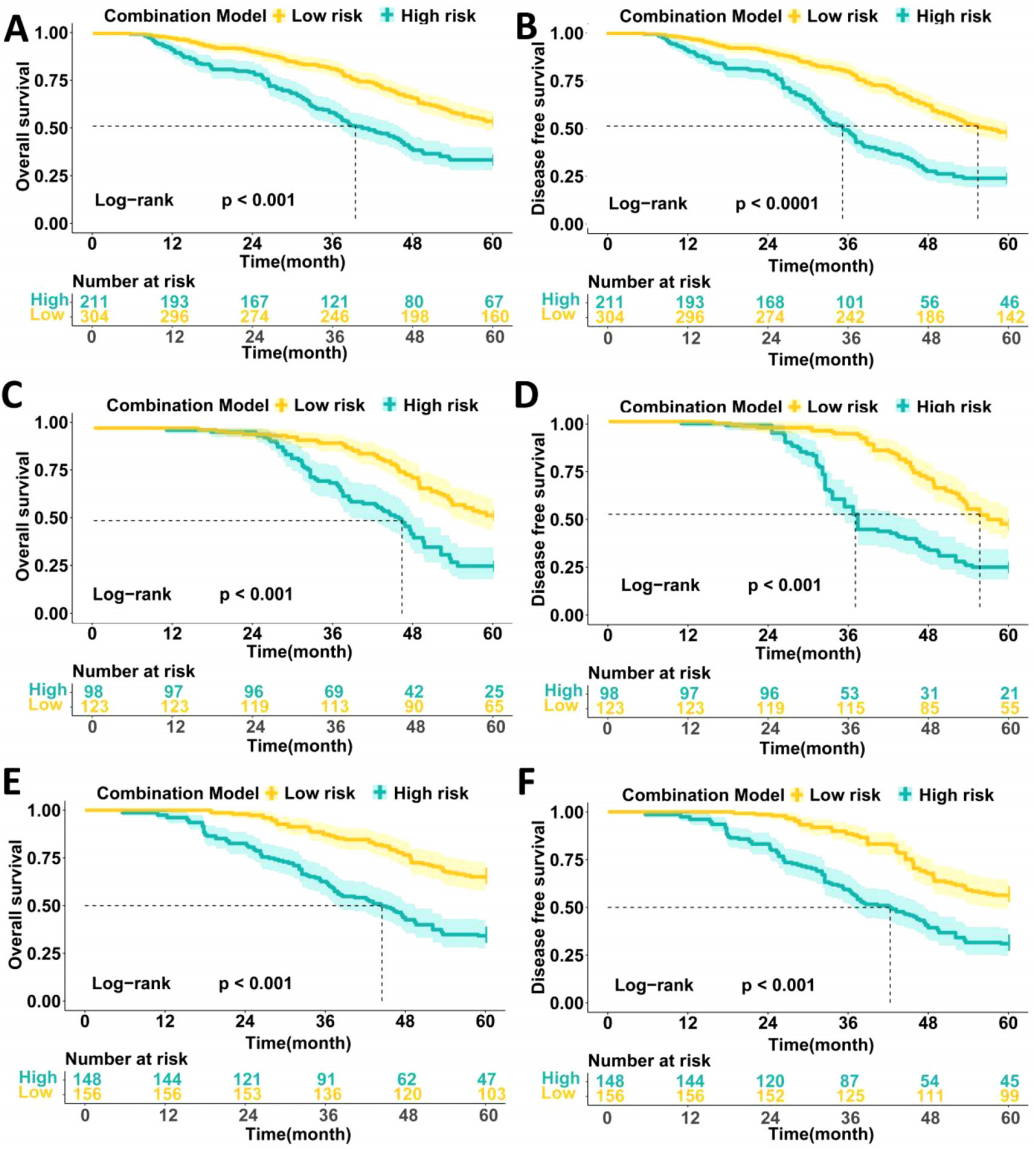
Comparison of the survival prognosis of LAGC patients in three data sets based on high and low expression groups of peripheral blood metabolic scores. **(A)** Comparison of 5-year OS survival curves of patients with high and low metabolic score groups in the training set; **(B)** Comparison of 5-year DFS survival curves of patients with high and low metabolic score groups in the training set; **(C)** Comparison of 5-year OS survival curves of patients with high and low metabolic score groups in the internal validation set; **(D)** Comparison of 5-year DFS survival curves of patients with high and low metabolic score groups in the internal validation set; **(E)** Comparison of 5-year OS survival curves of patients with high and low metabolic score groups in the external validation set; **(F)** Comparison of 5-year DFS survival curves of patients with high and low metabolic score groups in the external validation set.

We followed up on 1,040 LAGC patients included in the analysis, evaluating survival outcomes across three datasets. In the training set, patients with high metabolic scores had significantly worse 5-year OS (33.0% vs. 53.0%, P < 0.0001) and DFS (22.6% vs. 47.7%, P < 0.0001) compared to those with low metabolic scores (**Figures 4A, B**). Similar results were observed in both the internal and external validation sets, where high metabolic score patients showed poorer 5-year OS (internal validation: 25.0% vs. 52.8%, P < 0.0001; external validation: 33.5% vs. 65.1%, P < 0.0001) and DFS (internal validation: 20.8% vs. 44.8%, P < 0.0001; external validation: 31.0% vs. 56.4%, P < 0.0001) (**Figures 4C–F**).

Cox multivariable analysis revealed that TNM stage, pathological type, and metabolic score are independent prognostic factors for 5-year OS in locally advanced gastric cancer patients. In univariate analysis, patients with stage III disease had a significantly higher risk than those with stages I/II (HR = 4.469, P < 0.001), while patients with high pathological type had a greater survival risk compared to those with low/no pathological type (HR = 3.323, P = 0.002). Patients with high metabolic scores had a significantly shorter survival compared to those with low scores (HR = 4.245, P = 0.001). Multivariable analysis further confirmed TNM stage (HR = 5.223, P < 0.001), pathological type (HR = 2.423, P = 0.011), and metabolic score (HR = 3.785, P = 0.001) as independent prognostic factors (**Table 6**).

**Table 6:** Cox multivariable analysis of factors affecting 5-year OS in patients with locally advanced gastric cancer.

Variable	Univariable Analysis		Multivariable Analysis	
	HR (95% CI)	*P*	HR (95% CI)	*P*
Gender		0.541		
Male	Reference			
Female	0.872(0.432–1.761)			
Age(years)		0.346		
≤65	Reference			
>65	0.734(0.236–1.233)			
TNM stage		0.000		0.000
I/II	Reference		Reference	
III	4.469(2.148–8.437)		5.223(2.788–9.877)	
Primary site		0.218		
Up 1/3	Reference			
Middle 1/3	0.432(0.248–2.157)			
Lower 1/3	0.576(0.251–1.322)			
Tumor size(cm)		0.028		0.079
≤5	Reference		Reference	
>5	1.891(1.239–3.674)		1.211(0.679–3.214)	
Histology		0.002		0.011
High	Reference		Reference	
None/Low	3.323(1.679–5.674)		2.423(1.569–4.124)	
Nerve invasion		0.017		0.327
No	Reference		Reference	
Yes	1.674(1.423–3.675)		1.214(0.763–2.985)	
Vascular invasion		0.444		
No	Reference			
Yes	0.609(0.123–3.342)			
Lauren		0.041		0.231
Intestinal	Reference		Reference	
Mixed	1.521(1.128–3.225)		1.221(0.342–2.895)	
Metabolic score		0.001		0.001
Low	Reference		Reference	
High	4.245(2.348–7.218)		3.785(2.238–6.567)	

Additionally, Cox multivariable analysis demonstrated that TNM stage, pathological type, and metabolic score are independent prognostic factors for 5-year DFS. In univariate analysis, patients with stage III disease had a significantly higher risk of recurrence compared to those with stages I/II (HR = 5.234, P = 0.001), while patients with high pathological type had a higher risk of recurrence compared to those with low/no pathological type (HR = 2.454, P = 0.013). Patients with high metabolic scores had a significantly higher risk of recurrence than those with low scores (HR = 4.521, P = 0.001). In multivariable analysis, TNM stage (HR = 5.784, P = 0.001) and metabolic score (HR = 4.231, P = 0.001) remained independent prognostic factors, while pathological type (HR = 2.674, P = 0.003) continued to show significant prognostic impact (**Table 7**).

**Table 7:** Cox multivariable analysis of factors affecting 5-year DFS in patients with locally advanced gastric cancer.

Variable	Univariable Analysis		Multivariable Analysis	
	HR (95% CI)	*P*	HR (95% CI)	*P*
Gender		0.378		
Male	Reference			
Female	0.442(0.256–1.631)			
Age(years)		0.632		
≤65	Reference			
>65	0.563(0.124–1.263)			
TNM stage		0.001		0.001
I/II	Reference		Reference	
III	5.234(2.568–9.337)		5.784(2.568–8.897)	
Primary site		0.236		
Up 1/3	Reference			
Middle 1/3	0.562(0.318–1.237)			
Lower 1/3	0.728(0.251–1.902)			
Tumor size(cm)		0.022		0.562
≤5	Reference		Reference	
>5	1.743(1.139–3.674)		0.895(0.679–1.862)	
Histology		0.013		0.003
High	Reference		Reference	
None/Low	2.454(1.239–4.343)		2.674(1.272–5.123)	
Nerve invasion		0.027		0.267
No	Reference		Reference	
Yes	1.564(1.123–2.458)		1.144(0.783–3.098)	
Vascular invasion		0.044		0.364
No	Reference		Reference	
Yes	1.269(1.123–2.322)		1.189(0.893–1.892)	
Lauren		0.010		0.342
Intestinal	Reference		Reference	
Mixed	1.421(1.144–3.565)		1.091(0.674–2.785)	
Metabolic score		0.001		0.001
Low	Reference		Reference	
High	4.521(2.457–8.565)		4.231(2.787–7.762)	

## Discussion

In this study, we developed and validated a predictive model based on four peripheral blood metabolic markers (LHR, TCHR, TGHR, and TyG) to assess postoperative recurrence risk in patients with locally advanced gastric cancer (LAGC). The results revealed that high levels of these metabolic markers were significantly associated with increased recurrence risk and worse survival outcomes, both in terms of OS and DFS. Furthermore, multivariable Cox regression analysis identified these metabolic markers, along with TNM stage and pathological type, as independent prognostic factors. The novel metabolic score, combining these markers, showed robust predictive capabilities, outperforming clinical features alone in the prediction of recurrence. This model also demonstrated strong predictive performance across different datasets (training, internal, and external validation sets), with excellent accuracy, sensitivity, and specificity, indicating its potential for use in clinical practice to guide postoperative management in LAGC patients.

Previous studies have emphasized the importance of metabolic alterations in cancer prognosis (25). Several studies have investigated the role of individual metabolic markers, such as the TyG, in predicting the prognosis of GC). For example, a study by Kim et al. demonstrated that TyG was significantly associated with poor prognosis and recurrence in gastric cancer patients (26). Similarly, other studies have shown that lipid ratios, such as the low-density LHR correlate with the prognosis of various cancers, including gastric cancer (27, 28). LHR has been found to reflect not only lipid metabolism but also inflammation and oxidative stress, which are known to play key roles in tumor progression (29). In contrast to previous studies that focused on single metabolic markers, our study integrated multiple metabolic indices into a composite score, which significantly improved predictive accuracy for recurrence. This aligns with findings from recent research in other cancer types, such as colorectal and breast cancer, where combining multiple biomarkers provided a more comprehensive risk stratification (30, 31).

While individual metabolic markers have been widely studied, the integration of multiple markers into a composite score is a novel approach that offers several advantages. One of the strengths of our model lies in its ability to incorporate diverse aspects of metabolic dysregulation. Metabolic alterations, including lipid and glucose metabolism, insulin resistance, and inflammation, are known to interact in complex ways and contribute to cancer progression. Our findings support this notion by demonstrating that high levels of multiple metabolic markers, including LHR, TCHR, TGHR, and TyG, are independently associated with worse prognosis. This composite score reflects the multifactorial nature of metabolic disruption in cancer and may better capture the metabolic environment that promotes recurrence. This is particularly important for cancers like gastric cancer, where metabolic changes can influence not only tumor growth but also the tumor microenvironment and immune responses (32–35). By integrating these markers, we offer a more holistic approach to recurrence prediction, which could be more reliable than using individual markers alone.

From a molecular perspective, the results of this study highlight the significant role that metabolic dysregulation plays in cancer progression and recurrence. Metabolic markers like LHR, TCHR, TGHR, and TyG are linked to various underlying molecular mechanisms, including lipid metabolism, insulin resistance, and systemic inflammation, which can directly or indirectly promote tumor growth. For instance, the LHR ratio reflects dyslipidemia, a condition frequently observed in cancer patients. Dyslipidemia has been associated with the increased availability of fatty acids, which fuel tumor cell proliferation and metastasis. Elevated triglycerides and low HDL levels have also been implicated in promoting inflammation and endothelial dysfunction, both of which contribute to tumor angiogenesis and metastasis (36). Furthermore, the TyG index, which combines triglyceride and fasting blood glucose levels, has been linked to insulin resistance, a known driver of cancer progression (37, 38). Together, these metabolic alterations provide a favorable microenvironment for cancer cell survival, migration, and resistance to therapy. Therefore, our findings suggest that a metabolic composite score based on these markers not only predicts recurrence but also reflects underlying molecular processes that contribute to cancer progression. By combining multiple indicators, it is possible to capture the complex changes in the metabolic system more comprehensively, changes that may not be revealed by a single indicator.

Despite the promising findings, this study has several limitations that should be addressed in future research. One limitation is the retrospective nature of the study, which inherently introduces bias due to the use of historical data. Although we have minimized the impact of biases through strict data filtering and multi-center validation, these limitations may still affect the credibility of the model. While we validated the model across different datasets, prospective validation is needed to confirm the predictive power and generalizability of the composite score in real-world clinical settings. Additionally, while the study focused on peripheral blood markers, other factors, such as genetic, epigenetic alterations and gut microbiota, may provide a more comprehensive perspective on recurrence risk. Integrating these factors with metabolic markers may enhance the model’s predictive accuracy and provide a more comprehensive understanding of the molecular mechanisms driving recurrence in LAGC patients. Furthermore, the study did not explore the impact of adjuvant therapies, which may interact with metabolic markers and influence recurrence risk. Future studies should consider incorporating these variables to refine the predictive model further. Additionally, the lack of complete postoperative dynamic monitoring data for peripheral blood metabolic composite scores posed a challenge for conducting a unified analysis of postoperative trends. Addressing this limitation in future research by incorporating comprehensive postoperative monitoring could offer deeper insights.

In conclusion, our study highlights the potential of a peripheral blood metabolic composite score based on LHR, TCHR, TGHR, and TyG as a reliable and practical tool for predicting postoperative recurrence in LAGC patients. The model demonstrated strong predictive power across multiple validation cohorts and was found to outperform traditional clinical features alone. Given its non-invasive nature and the widespread availability of the required biomarkers, this composite score could serve as a valuable tool for clinicians to identify high-risk patients and guide postoperative management decisions. As we continue to refine the model and validate it prospectively, it has the potential to become an integral part of personalized treatment strategies for LAGC patients, ultimately improving clinical outcomes and survival.

## Data Availability

The raw data supporting the conclusions of this article will be made available by the authors, without undue reservation.
