# Supplementary material for: Peripheral blood metabolic composite score based on peripheral blood metabolism can be used as an assessment of recurrence after surgery in patients with locally advanced gastric cancer: a novel and promising index
Source: Front Oncol. 2025 Apr 17;15:1536811. doi: 10.3389/fonc.2025.1536811 (PMC12043443; doi:10.3389/fonc.2025.1536811)
Supplement: Supplementary file 1 [file Table1.docx]

**Supplementary Table 1 Comparison of preoperative and 7 days after surgery of four peripheral blood metabolic indexes**

| **Variable** | **LHR** | | | **P** | | **TCHR** | | | **P** | | **TGHR** | | | **P** | | **TyG** | | | **P** | |
| --- | --- | --- | --- | --- | --- | --- | --- | --- | --- | --- | --- | --- | --- | --- | --- | --- | --- | --- | --- | --- |
|  | **High** | **Low** |  | | **High** | | **Low** |  | | **High** | | **Low** |  | | **High** | | **Low** |  | |  |
| preoperative | 464(44.6%) | 576(55.3%) | 0.309 | | 510(49.1%) | | 530(50.9%) | 0.236 | | 474 (45.3%) | | 566 (54.4%) | 0.597 | | 429 (41.3%) | | 611 (58.7%) | 0.689 | |  |
| 7 days after surgery | 441(42.4%) | 599(57.6%) |  |  | 483(46.4%) | | 557(53.6%) |  |  | 462 (44.4%) | | 578 (55.6%) |  |  | 438 (42.1%) | | 602 (57.9%) |  |  |  |

**Supplementary Table 2 preoperative and 7 days after surgery peripheral blood metabolic indexes for predicting recurrence of LOGC**

|  | **AUC (95%CI)** | | | **DeLong test** |
| --- | --- | --- | --- | --- |
|  | **preoperative** | **7 days after surgery** | ***p*** | |
| **Metabolic score** |  |  |  | |
| Training set | 0.821(0.785-0.858) | 0.809(0.771-0.846) | 0.057 | |
| Internal validation set | 0.814(0.756-0.872) | 0.798(0.738-0.858) | 0.118 | |
| External validation set | 0.824 (0.778-0.870) | 0.811 (0.763-0.859) | 0.177 | |
| **Combination Model** |  |  |  | |
| Training set | 0.867 (0.836-0.897) | 0.858 (0.826-0.889) | 0.021 | |
| Internal validation set | 0.887 (0.844-0.929) | 0.874 (0.828-0.918) | 0.056 | |
| External validation set | 0.859 (0.817-0.899) | 0.847 (0.804-0.891) | 0.029 | |
